# Transcript profile of skeletal muscle lipid metabolism genes affected by diet in a piglet model of low birth weight

**DOI:** 10.1371/journal.pone.0224484

**Published:** 2019-10-29

**Authors:** Miriama Sciascia, Gürbüz Daş, Steffen Maak, Claudia Kalbe, Barbara U. Metzler-Zebeli, Cornelia C. Metges

**Affiliations:** 1 Institute of Nutritional Physiology, Leibniz Institute for Farm Animal Biology, Wilhelm-Stahl-Allee, Dummerstorf, Germany; 2 Institute of Muscle Biology and Growth, Leibniz Institute for Farm Animal Biology, Wilhelm-Stahl-Allee, Dummerstorf, Germany; 3 Institute of Animal Nutrition and Functional Plant Compounds, Department for Farm Animals and Veterinary Public Health, University of Veterinary Medicine Vienna, Austria; INIA, SPAIN

## Abstract

Dysregulated skeletal muscle metabolism (DSMM) is associated with increased inter- and intramuscular fat deposition in low birth weight (L) individuals. The mechanisms behind DSMM in L individuals are not completely understood but decreased muscle mass and shifts in lipid and carbohydrate utilisation may contribute. Previously, we observed lower fat oxidation in a porcine model of low birth weight. To elucidate the biological activities underpinning this difference microfluidic arrays were used to assess mRNA associated with lipid metabolism in longissimus dorsi (LD) and semitendinosus (ST) skeletal muscle samples from thirty-six female L and normal birth weight (N) pigs. Plasma samples were collected from a sub-population to measure metabolite concentrations. Following overnight fasting, skeletal muscle and plasma samples were collected and the association with birth weight, diet and age was assessed. Reduced dietary fat was associated with decreased LD intermuscular fat deposition and beta-oxidation associated mRNA, in both birth weight groups. Lipid uptake and intramuscular fat deposition associated mRNA was reduced in only L pigs. Abundance of ST mRNA associated with lipolysis, lipid synthesis and transport increased in both birth weight groups. Lipid uptake associated mRNA reduced in only L pigs. These changes were associated with decreased plasma L glucose and N triacylglycerol. Post-dietary fat reduction, LD mRNA associated with lipid synthesis and inter- and intramuscular fat deposition increased in L, whilst beta-oxidation associated mRNA remains elevated for longer in N. In the ST, mRNA associated with lipolysis and intramuscular fat deposition increased in both birth weight groups, however this increase was more significant in L pigs and associated with reduced beta-oxidation. Analysis of muscle lipid metabolism associated mRNA revealed that profile shifts are a consequence of birth weight. Whilst, many of the adaptions to diet and age appear to be similar in birth weight groups, the magnitude of response and individual changes underpin the previously observed lower fat oxidation in L pigs.

## Introduction

Skeletal muscle is the primary metabolic organ for the disposal of lipids (and carbohydrates) and energy dissipation, where it plays a central role in regulating whole body energy homeostasis [1]. The regulation of lipid metabolism is a complex process that exists in a cycle of uptake, storage (lipid synthesis: when exogenous energy supply is in excess), release (lipolysis: when exogenous energy supply is not sufficient) and energy generation (beta-oxidation: when there is a demand for ATP) [1]. Dysregulation of these processes has been linked to the development of metabolic disorders such as obesity and type 2 diabetes, which have chronic negative impacts on an individual’s health and development [2]. A well-regarded model for investigating the etiology of metabolic disorders is the low birth weight (L) pig [3].

The skeletal muscles of L pigs have been shown to be prone to increased intramuscular (IMF; also known as intra-myocellular lipids) and intermuscular fat (INTMF; extra-myocellular lipids) deposition before [3] and after [4] weaning. The presence of increased IMF and INTMF has been associated with the development of type 2 diabetes, insulin resistance and dysregulated skeletal muscle metabolism in humans [5]. Differences in IMF and INTMF deposition have been linked to lower fibre number; primarily secondary fibres, in L compared to normal birth weight (N) individuals [6, 7]. Low fibre number results in the plateau of fibre growth being attained earlier, thus, energy may no longer be partitioned towards muscle accretion, but instead it may be primarily used to support fat deposition [8]. Master regulators of muscle energy metabolism are the mitochondria, who regulate fat utilisation via beta-oxidation and storage via acetyl-CoA production from carbohydrate oxidation [9]. Thus, as fibre number and / or size changes, due to birth weight [10], breed [11] so does the ability of skeletal muscle to metabolise fats (and carbohydrates).

Our previous work showed that prior (75 days (d) of age) to a reduction in dietary fat (9.2 to 3.8%) at 80 d there was no difference in whole body fat (FOX) or carbohydrate oxidation (COX), between L and N pigs [12]. Post-change, at 83, 97 and 104 d, FOX was lower and COX was higher in L pigs, indicating a preference for carbohydrates as an energy source and earlier lipid deposition. Evidence of earlier lipid deposition has been previously reported by our group, however the mechanisms and the role of skeletal muscle was not investigated [13]. Two of the most intensively studied skeletal muscles are the longissimus dorsi (LD) and semitendinosus (ST), and in a recent study it has been shown that IMF accumulation occurs earlier and deposition amounts are greater in the LD compared to the ST, but glucose consumption was lower [14].

The hypothesis of our candidate gene approach was that L pigs are prone to a more pronounced transcription of genes involved with lipid uptake and synthesis. Consequently, to understand the potential role skeletal muscle plays in regulating whole body FOX and how birth weight affects these processes, the objectives of this study were: i) to compare transcript profile changes of lipid metabolism in the LD and ST muscle of L and N pigs, and ii) investigate transcript profile changes of lipid metabolism before and after the adaptation period to a reduction in dietary fat.

## Materials and methods

### Animal handling

Ethical approval for all experimental procedures were approved by the relevant authorities of Mecklenburg-Vorpommern, Germany (Landesamt fur Landwirtschaft, Lebensmittelsicherheit und Fischerei, Mecklenburg-Vorpommern; permission no. LALLF M-V/TSD/7221.3–1.1-049/09). All work was conducted in accordance with the German Animal Protection regulations.

Pure German Landrace pigs were sourced from and the following experiment conducted at the experimental pig facility of the Leibniz Institute for Farm Animal Biology. Thirty-four litters (2^nd^ to 7^th^ parity sows; mean live litter size *n* = 15 ± 0.4; mean live piglet weight = 1.3 ± 0.02; S1 Table) were used. At birth, pairs of female littermates were selected of which one had a low (L; 0.9–1.2 kg) and the other had a normal (N; 1.4–1.8 kg) birth weight. In this study, L piglets were defined as having a bodyweight less than the lower quartile of the average litter birth weight in our pig breeding facility. Females were chosen for this study to remove the influence of gender specific tissue development. Experimental animals were suckled by their dam, weaned at 28 d and fed ad libitum a post-weaning diet (S2 Table) with 16.9 MJ ME/kg of dry matter (15.9% crude protein, 33% starch, 27% sugar and 9.2% crude fat/kg) until 79 d (S3 Table). From 80 d onwards, pigs were fed a diet containing 15.2 MJ ME/kg of dry matter (16.5% crude protein, 44% starch, 14.8% sugar and 3.8% crude fat/kg). All pigs received identical diets and received two equal meals per day (0800 and 1500 h) as previously published [12]. All diets were formulated to supply energy and nutrients at or above recommendations, as published previously [12]. The experiment was performed with four different age groups (75, 98, 104, 131 d; trial design S3 Table) spread across seven different dates (blocks). At 74, 83, 96 d (*n* = 36/age group) and 113 d (*n* = 18) of age a subpopulation of experimental animals were fasted for 14 h, for the analysis of plasma metabolite profiles. At 75 (*n* = 20), 98 (*n* = 18), 104 (*n* = 18) and 131 (*n* = 16) d, following a 12 h fast, animals were euthanized and the skeletal muscles LD (between the 13th and 14th rib) and ST (from the central portion of the muscle midbelly) were snap frozen in liquid nitrogen and stored at -80 ^o^C for subsequent analysis.

### Plasma metabolites

Plasma samples (F-EDTA, Sarstedt AG & Co., Nümbrecht, Germany) were analysed for concentrations of non-esterified fatty acids (NEFA; number 434-91795s, Wako Chemicals GmbH, Neuss, Germany), glucose (no. 553–230) from MTL Diagnostics (Idstein, Germany), TG (GPO-PAP, number LT-TR 0015; Labor + Technik Lehmann, Berlin, Germany), urea (no. LT-UR 0050) and cholesterol (CHOD-PAP, no. LT-GL 01039) by the Clinic for Cattle (University of Veterinary Medicine Hannover, Hannover, Germany). Analyses were performed by spectrophotometry (Pentra 400, Axon Lab, Reichenbach, Germany).

### Microfluidic real-time PCR

#### RNA extraction, purification and cDNA synthesis.

Total RNA was extracted from pre-weighed -80 ^o^C frozen LD and ST tissue using peqGOLD TriFast (VWR International GmbH, Hannover, Germany), purified using RNeasy minikits (Qiagen, Hilden, Germany) and quantified using a Nanophotometer (Implen GmbH, Munich, Germany). The RNA quality was assessed using a Bioanalyzer 2100 and RNA 6000 Nano kit (Agilent Technologies, Waldbronn, Germany), with a RIN cutoff of 8.5. Synthesis of cDNA was performed using 250 ng of purified RNA and the reverse transcription master mix kit (Fluidigm, Amsterdam, Netherlands), according to the manufacturer’s instructions.

#### Primers and primer specific cDNA pre-amplification.

DELTAgene assays (Fluidigm) were designed for 27 lipid metabolism associated transcripts, and grouped according to function as shown in S4 Table. Briefly, five functional groups were created Lipid uptake / Transport (*n* = 3), Lipid Synthesis (*n* = 11), Lipolysis (*n* = 3), Beta-oxidation (*n* = 6), Nuclear receptors (*n* = 3) and Energy regulation (*n* = 1). Primers were designed using publicly available Sus scrofa transcript data available from NCBI (www.ncbi.nlm.nih.gov) and the primer design programs GenScript (https://www.genscript.com) or the Roche Universal ProbeLibrary assay design centre (http://www.roche-applied-science.com/). All primers (Metabion GmbH, Martinsried, Germany) were designed to be intron spanning and expected to behave identically during the pre-amplification. Pre-amplification was performed for 10 cycles using the PreAmp Master Mix (Fluidigm), according to the manufacturer’s instructions. For each assay, 1 μL of 100 μM forward and reverse primer was mixed into a tube and made to 200 μL with DNA suspension buffer (TEKnova, USA).

#### DELTAgene assay.

Pre-amplified cDNA samples were analysed by qPCR using 48.48 Dynamic Array^™^ integrated fluidic circuits (IFCs) and the BioMark™ HD System from Fluidigm. Processing of the IFCs and operation of the instruments were performed according to the manufacturer’s instructions. Each experiment consisted of analysing 15-duplicate experimental samples (plus three additional samples: an inter-run calibrator, a no-template and a no-enzyme control for qPCR), with 48 DELTAgene assays. In order to prepare samples for loading into the IFC, a mix was prepared consisting of 180 μL Sso Fast EvaGreen Supermix with Low ROX (BioRad 1725211), 18 μL 20× DNA Binding Dye Sample Loading Reagent (Fluidigm 1003738) and 3.3 μL of this mix was dispensed to each well of a 96-well assay plate (Sarstedt 72.1978.202). Pre-amplified cDNA (2.7 μL) was added to each well and the plate briefly vortexed and centrifuged. Following priming of the IFC in the IFC Controller HX, 5 μL of the cDNA mix was dispensed to each Sample Inlet of the 48.48 IFC. For the DELTAgene assays, 5 μL of each 10× Assay (5 μM each primer) were dispensed to each Detector Inlet of the 48.48 IFC. After loading the assays and samples into the IFC in the IFC Controller HX, the IFC was transferred to the BioMark HD and PCR was performed using the thermal protocol GE Fast 48 × 48 PCR + Melt v2.pcl. This protocol consists of a Hot Start at 95°C, 60 s, PCR Cycle of 40 cycles of (96°C, 5 s; 60°C, 20 s), and Melting using a ramp from 60°C to 95°C at 1°C/3 s.

#### Differential mRNA abundance analysis.

Data was analysed using the Fluidigm Real-Time PCR Analysis software using the Linear (Derivative) Baseline Correction Method and the Auto (Global) Ct Threshold Method and exported to qBASEplus 2.0 (Biogazelle, Ghent, Belgium). An upper limit Cq threshold of 31 cycles was set to remove poorly amplifying or low abundance cDNA targets, resulting in the LD targets *ACAA2*, *CPT1A*, *DGAT2*, *FABP4*, *FASN*, *GPAT1* and *GPAT3*, and the ST targets *DGAT2*, *FASN*, *GPAT1* and *GPAT3* being removed from the study, in addition to 2 pairs of animals. The reference gene 18S ribosomal RNA was included as a target and it appears that the extremely high abundance saturated the pre-amplification process, leading to some key targets with quantitation cycle (Cq) values over 31. Further analysis of the imported qPCR data showed no amplification for one pig in all LD and ST samples, so it and the littermate were removed from the study. Thus, the total number of samples analysed per tissue, and per time point were at 75 (*n* = 18), 98 (*n* = 16), 104 (*n* = 16) and 131 (*n* = 16) d. The GeNorm applet selected the reference genes; ST: *Hprt*, *Hspcb*, *Top2b*; LD: *Psmc3*, *Hprt*, *Hspcb*, out of five genes (*Hprt*, *Hspcb*, *Psmc3*, *Top2b*, *Rps18*) as the most stably expressed across the two birth weight groups and four time points used in this study (132 samples, all examined in duplicate). Reference genes were used to normalize target gene expression in the qBASEplus software and the Cq values were converted into Log-Calibrated Normalized Relative Quantity (CNRQ) values, taking into account amplification efficiencies, inter-run variations, and normalization factors [15]. All qPCR data were reported according to the published MIQE guidelines [16].

### Statistical analysis

Body weight and anthropometric data were analysed using the MIXED procedure of SAS (version 9.4; SAS Institute Inc., Cary, NC, USA) with the fixed factors of birth weight group (L, N) and sow as a random factor, with the repeated factor time point with the power spatial covariance matrix, used only for body weight analysis. Sow as a random allowed modelling of the potential dependency of littermates from the same sow. Plasma metabolite profiles were investigated with same model used to analyse pig body weight, but with the repeated factor of time of blood sampling (74, 83, 96 and 113 d). Probability values ≤ 0.05 were considered statistically significant, and values > 0.05 but ≤ 0.10 are discussed as trends. Real-time PCR Log-CNRQ values exported from qBASEplus 2.0 (Biogazelle, Ghent, Belgium) were statistically investigated using the MIXED procedure of SAS. The model included birth weight group (L, N), time point (75, 98, 104 and 131 d) and their interaction as fixed factors, experimental block (1, 2, 3, 4, 5, 6, and 7) as a factor, and the sow as a random factor. Probability values ≤ 0.05 were considered statistically significant, and values > 0.05 but ≤ 0.15 are discussed as trends. For all analyses, post hoc comparisons were performed using the Tukey–Kramer test. Results are reported as least square means (LSM) ± SE.

## Results and discussion

Dysregulated skeletal muscle metabolism (DSMM) has been observed in a number of low birth weight models, resulting from maternal undernutrition [17] and metabolic disorder [18], placental insufficiency [19], and natural occurrence [20]. A consequence of DSMM is increased adiposity potentiated by a reduction in the utilisation of fatty acids as an energy source and their shuttling towards lipogenesis. However, the transcriptional mechanisms regulating lipid metabolism in the skeletal muscle of L individuals during the transition to a diet lower in fat are not well characterised. To our knowledge, this is the first time that the lipid metabolism transcript profile has been analysed in two muscle groups during this transition and the subsequent adaptation period, in a low birth weight model.

### Body morphology and plasma metabolites

Prior to the dietary change L pigs were lighter (*P* < 0.01; Table 1), however, post change no difference in body weight was observed. Over this transition period is when we observed FOX and COX began to differ between L and N pigs [12]. In order to evaluate the potential effects of birth weight, diet and the post-diet change adaptation had on the metabolic status of L and N pigs, TAG, cholesterol, non-esterified fatty acid (NEFA), glucose and urea were measured. Plasma metabolite concentrations did not differ among L and N pigs at every time point measured. However, a comparison of the post- (83 d) to pre-dietary (74 d) time points, showed a significant decrease in plasma urea and cholesterol in both L and N pigs (Table 2). Plasma urea is a marker for amino acid utilization by protein synthesis, thus it appears that post dietary change, both L and N pigs had improved protein synthesis. The lower cholesterol concentration might reflect the lower fat intake. No changes in plasma NEFA concentrations were observed for either birth weight group. Interestingly, data analysis showed significantly lower plasma glucose concentrations in L pigs and a trend for lower plasma TAG in N pigs (Table 2), potentially reflecting differential energy substrate utilization between L and N pigs. The primary organ for the disposal of lipids (and carbohydrates) is the skeletal muscle, and thus transcriptional changes in lipid metabolism associated genes may explain the observed changes.

**Table 1:** Body weight (BW) and anatomical data from birth until age 131 days of low (L) and normal (N) birth weight pigs.

		Birth weight group			
Item	*n*	L	N	SE	*P value*
Birth weight, kg	72	1.09	1.52	0.020	*<0*.*001*
Anatomical data	72				
Crown-rump length, cm		23.85	26.54	0.210	*<0*.*001*
Birth weight/crown-rump length, g/cm		45.68	57.56	0.000	*<0*.*001*
BMI, kg/m2		19.18	21.74	0.300	*<0*.*001*
Ponderal index, kg/m3		80.71	82.33	1.750	0.47
BW, kg					
7 d	72	2.09	2.98	0.060	*<0*.*001*
14 d	72	3.55	4.96	0.120	*<0*.*001*
21 d	72	5.09	6.87	0.190	*<0*.*001*
27 d	72	6.43	8.42	0.230	*<0*.*001*
75 d	56	22.05	25.19	0.740	*<0*.*01*
98 d	36	34.20	36.62	1.390	0.12
104 d	27	37.89	42.07	2.130	0.13
131 d	16	67.48	69.46	2.670	0.61

Data are presented as least square means and their standard error (SE)

**Table 2:** Plasma metabolite concentrations at 74, 83, 96 and 113 d of age for a sub-population of low (L) and normal (N) birth weight (BiW) piglets used in this study.

		Birth weight group			*P value* ^1^		*P value* ^2^	
Metabolite^1^	*n*	L	N	SE	BiW	Day	L	N
Triacylglycerol (mmol/l)								
74 d	36	0.50	0.52	0.04	0.52	*<0*.*001*		
83 d	36	0.41	0.42	0.04	0.90		0.20	0.07
96 d	36	0.44	0.42	0.04	0.78		0.94	1.00
113 d	18	0.35	0.41	0.05	0.26		0.29	0.99
Cholesterol (mmol/l)								
74 d	36	2.82	2.72	0.08	0.35	*<0*.*001*		
83 d	36	2.58	2.50	0.08	0.49		0.01	0.03
96 d	36	2.38	2.44	0.08	0.58		0.11	0.88
113 d	18	2.45	2.46	0.11	0.99		0.92	1.00
NEFA (μmol/l)								
74 d	36	116.70	140.60	29.62	0.54	*<0*.*001*		
83 d	36	204.55	188.32	29.62	0.68		0.61	0.91
96 d	36	164.73	116.73	29.62	0.22		0.76	0.29
113 d	18	139.47	151.24	40.82	0.83		0.95	0.89
Glucose (mmol/l)								
74 d	36	6.46	5.96	0.22	0.12	*<0*.*001*		
83 d	36	5.21	5.53	0.22	0.31		0.00	0.55
96 d	36	5.52	5.61	0.22	0.79		0.77	1.00
113 d	18	5.33	5.38	0.32	0.92		0.96	0.94
Urea (mmol/l)								
74 d	36	2.33	2.39	0.42	0.82	0.18		
83 d	36	2.05	2.08	0.42	0.91		0.02	0.02
96 d	36	1.94	1.92	0.42	0.95		0.75	0.47
113 d	18	1.63	1.73	0.44	0.76		0.32	0.72

Data are presented as least square means and their standard error (SE)

^1^An interaction of BW x Day was only observed for plasma NEFA (*P <* 0.001)

^2^Comparsion of values from each subsequent time point to the preceding (74 vs. 83, 96 vs. 83 and 113 vs. 96)

### Longissimus dorsi, diet dependent adaptation

A comparison of post- (98 d) and pre- (75 d) dietary change showed that *PLIN2* (Perilipin 2: lipid synthesis; *P <* 0.01), *ATGL* (Adipose triglyceride lipase), *HSL* (Hormone-sensitive lipase), *MGLL* (Monoglyceride Lipase) (lipolysis; *P <* 0.01), *CPT1B* (Carnitine palmitoyltransferase 1B: beta-oxidation; *P <* 0.01), *PPARA* (Peroxisome proliferator-activated receptor alpha: nuclear receptor; *P <* 0.01) and *PGC1* (Peroxisome proliferator-activated receptor gamma coactivator 1-alpha: energy regulation; *P <* 0.01) mRNA were significantly lower at 98 d, in both L and N pigs (Table 3). These results indicate that both L and N pigs shifted energy away from IMF storage and reduced fatty acid (FA) oxidation in response to the change of diet at 80 d. In addition, at 98 d *FABP3* (Fatty acid-binding protein 3: lipid transport; *P <* 0.01) mRNA was significantly lower in only N pigs, whilst *PLIN1* (Perilipin 1: lipid synthesis; *P <* 0.01) was lower and *ACADL* (Acyl-CoA dehydrogenase. long chain: beta-oxidation; *P <* 0.01) mRNA was higher in only L pigs (Table 3). Thus, it appears FABP3-mediated FA trafficking was reduced in only N pigs, whilst PLIN1-associated INTMF deposition decreased in only L pigs.

**Table 3:** The effect of reduced dietary fat (pre-diet (75 days of age (d)) to post-diet change (98 d) time point comparison) and age (time point comparisons 98, 104 and 133 d; post-dietary fat reduction), on the abundance of longissimus dorsi mRNA molecules associated with lipid metabolism (lipid synthesis, lipolysis and beta oxidation) in low (L) and normal (N) birth weight pigs.

	L					*P value*		
mRNA molecules^1^	75 d	98 d	104 d	133 d	SE	75 v. 98 d	98 v. 104 d	104 v. 133 d
Lipid Synthesis								
*ACACA*	-0.20	-0.02	-0.07	0.77	0.148	0.83	0.99	*<0*.*01*
*PLIN1*	1.21	-0.90	-1.04	1.06	0.381	*<0*.*01*	0.98	*<0*.*01*
*PLIN2*	0.94	-1.38	-0.51	0.80	0.322	*<0*.*01*	0.04	0.01
Lipolysis								
*ATGL*	1.61	-0.73	-0.32	-0.48	0.437	*<0*.*01*	0.76	0.99
*HSL*	0.87	-0.35	-0.40	0.39	0.177	*<0*.*01*	0.99	*<0*.*01*
*MGLL*	0.99	-0.37	-0.15	-0.44	0.226	*<0*.*01*	0.83	0.72
Beta-oxidation								
*ACADL*	-0.55	0.31	-0.44	0.68	0.170	*<0*.*01*	*<0*.*01*	*<0*.*01*
*CPT1B*	1.26	-0.33	0.16	-1.46	0.221	*<0*.*01*	0.18	*<0*.*01*
Nuclear Receptors								
*PPARA*	0.44	-0.36	-0.22	0.43	0.184	*<0*.*01*	0.87	0.02
Energy Regulation								
*PGC1*	0.55	-0.34	-0.33	-0.28	0.270	*<0*.*01*	1.00	1.00
	N					*P value*		
mRNA molecules^1^	75 d	98 d	104 d	133 d	SE	75 v. 98 d	98 v. 104 d	104 v. 133 d
Lipid Transport								
*FABP3*	0.58	-0.29	0.09	-0.30	0.145	*<0*.*01*	0.12	0.14
Lipid Synthesis								
*PLIN1*	0.47	-0.63	-1.12	0.68	0.381	0.17	0.55	*<0*.*01*
*PLIN2*	0.80	-0.89	-0.62	0.72	0.322	*<0*.*01*	0.82	*<0*.*01*
Lipolysis								
*ATGL*	1.46	-1.14	-0.66	-0.51	0.437	*<0*.*01*	0.67	0.99
*HSL*	0.69	-0.64	-0.66	0.32	0.177	*<0*.*01*	1.00	*<0*.*01*
*MGLL*	1.04	-0.35	-0.28	-0.57	0.226	*<0*.*01*	0.99	0.71
Beta-oxidation								
*ACADL*	-0.13	0.21	-0.22	0.66	0.170	0.49	0.14	*<0*.*01*
*CPT1B*	1.26	-0.31	0.47	-1.53	0.221	*<0*.*01*	0.01	*<0*.*01*
*HADH*	-0.14	-0.16	0.54	-0.30	0.232	1.00	0.05	*<0*.*01*
Nuclear Receptors								
*PPARA*	0.39	-0.73	-0.37	0.36	0.184	*<0*.*01*	0.22	0.01
*PPARG*	-0.20	-0.14	-0.13	0.54	0.255	1.00	1.00	0.15
Energy Regulation								
*PGC1*	0.84	-0.64	-0.07	-0.13	0.270	*<0*.*01*	0.23	1.00

Data are presented as least square means and their standard error (SE). SE; the largest value is shown

^1^mRNA abundance values are Calibrated Normalized Relative Quantity (CNRQ) generated in qBASEplus 2.0. Only molecules with *P* values ≤ 0.15 are shown

When birth weight groups were compared, data analysis showed that pre-dietary change (75 d), *FABP3* (*P =* 0.03) and *ACADL* (*P =* 0.03) mRNA abundance was lower, whilst *LPL* (Lipoprotein lipase: lipid uptake; *P =* 0.05), *PLIN1* and *SCD* (Stearoyl-CoA desaturase) (lipid synthesis; *P <* 0.01 and *P =* 0.08 respectively) mRNA abundance was higher in L pigs (Table 4). Thus, prior to the dietary change lipid synthesis, potentially through LPL-mediated uptake of FA and MAG derived from the circulation, biosynthesis via SCD and deposition into INTMF appeared to be higher in L pigs. However, post-dietary change, INTMF deposition or abundance appeared to be significantly reduced in L pigs via an almost 2-fold decrease in *PLIN1* mRNA abundance (Table 4). Whilst *LPL* mRNA was no longer significantly higher in L pigs due to a 1.5-fold, increase in N pigs. In the same analysis *FABP3* (*P =* 0.10), SCD (*P =* 0.02), *HSL* (*P =* 0.06) and *PPARA* (nuclear-receptor; *P <* 0.01) mRNA abundance was higher in L pigs (Table 4).

**Table 4:** The effect of diet (75, 98 days of age (d)) and age (98, 104 and 133 d; post-dietary fat reduction) on the abundance of longissimus dorsi mRNA associated with lipid metabolism (lipid synthesis, lipolysis and beta-oxidation). Comparison between low (L) and normal (N) birth weight pigs.

mRNA	75 d				99 d				104 d				131 d			
Molecules^1^	L	N	SEM	*P value*	L	N	SEM	*P value*	L	N	SEM	*P value*	L	N	SEM	*P value*
Lipid Uptake / Transport																
*FABP3*	0.22	0.58	0.145	0.03	0.00	-0.29	0.121	0.10	-0.17	0.09	0.120	0.13	-0.31	-0.30	0.142	0.94
*LPL*	0.00	-0.10	0.046	0.05	0.05	-0.02	0.039	0.20	0.07	0.01	0.038	0.32	0.02	-0.03	0.045	0.36
Lipid Synthesis																
*ACACA*	-0.20	-0.30	0.148	0.51	-0.02	-0.05	0.125	0.85	-0.07	0.05	0.123	0.47	0.77	0.10	0.146	*<0*.*01*
*PLIN1*	1.21	0.47	0.381	*<0*.*01*	-0.90	-0.63	0.302	0.26	-1.04	-1.12	0.283	0.75	1.06	0.68	0.369	0.14
*SCD*	0.31	-0.12	0.225	0.08	0.11	-0.52	0.189	0.02	0.03	0.31	0.186	0.29	0.20	0.10	0.221	0.74
Lipolysis																
*HSL*	0.87	0.69	0.177	0.22	-0.35	-0.64	0.148	0.06	-0.40	-0.66	0.139	0.09	0.39	0.32	0.173	0.70
Beta Oxidation																
*ACADL*	-0.55	-0.13	0.170	0.03	0.31	0.21	0.143	0.62	-0.44	-0.22	0.141	0.26	0.68	0.66	0.167	0.91
*CPT1B*	1.26	1.26	0.221	0.98	-0.33	-0.31	0.182	0.91	0.16	0.47	0.169	0.07	-1.46	-1.53	0.216	0.68
Nuclear Receptors																
*PPARA*	0.44	0.39	0.184	0.66	-0.36	-0.73	0.147	*<0*.*01*	-0.22	-0.37	0.137	0.23	0.43	0.36	0.179	0.58

Data are presented as least square means and their standard error (SE)

^1^mRNA abundance values are Calibrated Normalized Relative Quantity (CNRQ) generated in qBASEplus 2.0. Only molecules with *P* values ≤ 0.15 are shown

Lipoprotein lipase is the rate-limiting enzyme for the uptake of FA by muscle and adipose tissue [21]. It is a transcriptionally, translationally and post-translationally regulated enzyme, which is synthesized and secreted by adipocytes and muscle to the capillary endothelium [22] where it hydrolyses the TAG core of circulating TAG-rich lipoproteins, chylomicrons, and VLDLs (Very-low-density lipoprotein) [23]. The hydrolysis products, FA and MAG, are then taken up by the target tissues to be stored or metabolized. Storage is regulated by the Perilipin family of proteins [24], of which PLIN1 is unique, as it is expressed only in adipocytes and steroidogenic cells [25], where the PLIN1 protein associates with lipid droplets to regulate INTMF deposition [26, 27]. The abundance of *PLIN1* mRNA has been used as a marker of INTMF content in the LD and semimembranosus muscles of pigs [26]. Thus, prior to the dietary change, the LD of L pigs appeared to favour INTMF lipid synthesis. A key enzyme in the pathways that utilize the dietary FA made available by LPL, or derived from lipolysis is stearoyl-CoA desaturase (SCD), which catalyses the rate-limiting step in the biosynthesis of monounsaturated fatty acids destined for storage in lipid droplets [28, 29]. In this study, the abundance of *SCD* mRNA was higher pre- and post-dietary change in L pigs, indicating endogenous synthesis of substrates required for lipid droplet formation/lipid was unaffected by the diet change. A higher abundance of *SCD1* mRNA has been shown to increase lipid accumulation in muscle [30, 31], by shifting substrates away from, and consequently lowering, beta-oxidation, as observed in this study. Pre-dietary change, the abundance of *HSL* and *PPARA* mRNA was higher in the LD of L pigs. Hormone sensitive lipase (HSL) is an enzyme that has been shown to hydrolyse TAG, DAG and MAG [32] and higher levels of *HSL* mRNA have been shown to lead to increased intracellular FA concentrations. These FA stimulate *Ppara* expression and bind to the protein product, facilitating its relocation to key genes involved in the regulation of FA metabolism, such as SCD. Thus, higher *SCD* mRNA, pre-dietary change, appears to be maintained by FA-activated PPARA, FA released through HSL mediated lipolysis, whilst the absence of higher *PPARA* mRNA post-dietary change suggests potential insulin-mediated regulation [33]. Interestingly, LPL expression is also regulated by insulin [34], and higher abundance of LPL and SCD has been linked to reduced beta-oxidation, and the development of hypertriglyceridemia and diabetes [30, 35]. The exact mechanisms are unknown, but what links them all is the accumulation of FA within the cell [29], their partitioning towards lipid droplet formation and the inhibitory properties these FA have on beta-oxidation [31]. Only one marker of beta-oxidation, pre- and post-dietary change was different between L and N pigs, *ACADL* (Acyl-CoA Dehydrogenase Long Chain), whose abundance was lower in L pigs, whilst none of the other investigated genes related to beta-oxidation were different. Fatty acid-binding protein (FABP3) is the major FABP in skeletal muscle [36] and several studies have shown that FABP3 is involved in FA transport, its oxidation or activation of nuclear-associated genes / proteins involved in energy homeostasis and fatty acid oxidation [37]. In this study, *FABP3* mRNA abundance was higher in the LD of N pig’s pre-dietary change; however, post-dietary change the abundance was higher in L pigs. Increased abundance of *FABP3* mRNA has been shown to increase glucose uptake independent of insulin signalling in C2C12 myotubes [38], potentially explaining why plasma glucose levels reduce significantly from the pre- to post-diet change period, in L pigs only.

### Longissimus dorsi, post diet adaptation

A time point comparison from 98 to 104 d showed there was increased *FABP3* (lipid transport; *P <* 0.12), *CPT1B* and *HADH* (beta-oxidation; *P =* 0.01, *P =* 0.05), and decreased *ACADL* (beta-oxidation; *P =* 0.14) mRNA in N pigs (Table 3). Whereas only *PLIN2* (lipid synthesis; *P =* 0.04) increased and *ACADL* (*P <* 0.01) mRNA decreased in L pigs (Table 3). Analysis of these changes suggested that beta-oxidation in N pigs and IMF deposition in L pigs increased. From 104 to 133 d increased *PLIN1*, *PLIN2* (lipid synthesis; L: *P <* 0.01; N = *P <* 0.01), *HSL* (lipolysis; L: *P <* 0.01; N: *P <* 0.01), *ACADL* (L: *P <* 0.01; N: *P <* 0.01), *PPARA* (nuclear-receptor; L: *P =* 0.02; N: *P =* 0.01) mRNA, and decreased *CPT1B* (L: *P <* 0.01; N: *P <* 0.01) was observed in both L and N pigs (Table 3). Thus, markers of IMF and INTMF deposition increased in both L and N pigs. Interestingly, the mRNA abundance of beta-oxidation marker *ACADL* increased whilst its companion marker *CPT1B* decreased. This dysregulation has only been observed in one previous study [39], but its basis is unknown and there is insufficient data in this study to speculate. In addition, only increased *PPARG* (Peroxisome proliferator-activated receptor gamma: nuclear receptor; *P =* 0.15) and decreased *FABP3* (*P <* 0.14) and *HADH* (*P <* 0.01) was observed in N pigs (Table 3). *PPARG* is primarily expressed in adipocytes where it regulates glucose uptake and fat storage [11], whereas *FABP3* is primarily expressed in muscle.

Comparative analysis of the birth weight groups showed that at 98 d *FABP3* (*P =* 0.10), *SCD* (lipid synthesis; *P =* 0.02), *HSL* (lipolysis; *P =* 0.06) and *PPARA* (nuclear receptor; *P <* 0.01) mRNA abundance was higher in L pigs (Table 4). By 104 d *FABP3* (*P =* 0.13) was now trending lower, and *HSL* (*P =* 0.09) and *CPT1B* (*P =* 0.07) mRNA were trending higher in L pigs. At 131 d *ACACA* (Acetyl-CoA carboxylase Alpha: lipid synthesis; *P <* 0.01) was and *PLIN1* (*P =* 0.14) tended to be higher in L pigs (Table 4). Thus, from 98 to 104 d L pigs appear to reduce lipolysis and beta-oxidation, and transition to increased INTMF deposition by 131 d, compared with their N littermates.

At 98 d, the LD of L pigs appears to be releasing IMF stores of FA in a negative feedback-loop that leads to the repression of beta-oxidation, and may explain why in our companion study FOX reduced from 75 to 98 d [12]. Whilst at 98 d no changes in the abundance of mRNA encoding proteins involved in beta-oxidation were observed, at 104 d the abundance of *CPT1B* was lower in L pigs. Carnitine Palmitoyltransferase 1B (CPT1B) is the muscle specific isoform of the protein required for the uptake of long chain fatty acids by the mitochondria, the first and rate-limiting step in beta-oxidation [40] and a well-established marker of beta-oxidation [41]. Murine studies using synthetic inhibitors [42] and knockouts [43] of Cpt1b in skeletal muscle have shown that inhibition or knockout leads to increased COX, utilization of amino acids as an energy source, and the accumulation of intracellular DAG and TAG [42, 43]. Whilst the intracellular amino acid and DAG / TAG content was not measured in this study, a companion study did show increased COX [12], suggesting that the LD of L pigs may contribute to the observed increase in whole body COX. This may also explain the reduced abundance of *FABP3* mRNA in L pigs at 104 d, which plays a role in the transport of FA destined for transport by CPT1B. Indeed, murine *Fabp3* knockout studies in the skeletal and heart muscle have shown that the uptake of FA was almost completely inhibited and tissue metabolism switched from FOX to COX. The higher abundance of HSL at 104 d is interesting given its role in the release of FA and MAG via the hydrolysis of DAG [32]. Diacylglycerols have been implicated in the development of insulin resistance in human skeletal muscle, resulting in reduced glucose clearance from circulation [44]. A higher abundance of HSL may provide a mechanism to prevent insulin resistance from developing in the LD of L pigs, by reducing the level of DAG and allowing for greater glucose clearance to support increased levels of COX [12]. By 131 d repression of beta-oxidation appears to be maintained in the LD of L pigs, via higher *ACACA* mRNA, and its association with malonyl-CoA repression of CPT1B activity and the channelling of FA-substrates to INTMF fat deposition via PLIN1.

### Semitendinosus, diet dependent adaptation

Analysis of the pre- (75 d) and post- (98 d) dietary change data showed that the abundance of *ATGL*, *HSL*, *MGLL* (lipolysis; L: *P =* 0.03, *P <* 0.03, P = 0.03; N: *P =* 0.13, *P <* 0.01, *P =* 0.08), *DGAT1* (Diacylglycerols acyltransferase 1), *PLIN2* (lipid synthesis; L: *P =* 0.10, *P =* 0.06; N: *P =* 0.03, *P =* 0.01) and *FABP3* (lipid transport; L: *P =* 0.15; N: *P =* 0.02) mRNA were higher, and *NR4A1* (Nuclear Receptor Subfamily 4, Group A Member 1: nuclear receptor; L: *P =* 0.03; N: *P =* 0.01) was lower, at 98 d, in both L and N pigs (Table 5). These changes are in contrast to the LD where the abundance of lipolysis and lipid synthesis markers was lower at 98 d, indicating muscle specific changes in lipid metabolism in response to the dietary change. Additionally, the data indicates that even though lipolysis may have increased in both L and N pigs so had IMF deposition and lipid transport. There were unique changes, but only in L pigs, where *LPL* was lower (lipid uptake; *P =* 0.01) and *FABP4* mRNA was higher (Fatty acid binding protein 4: lipid transport; *P =* 0.06) reflecting potentially lower FA uptake and increased transport compared to N pigs (Table 5).

**Table 5:** The effect of reduced dietary fat (pre-diet (75 days of age (d)) to post-diet change (98 d) time point comparison) and age (time point comparisons 98, 104 and 133 d; post-dietary fat reduction), on the abundance of semitendinosus mRNA molecules associated with lipid metabolism (lipid synthesis, lipolysis and beta oxidation) in low (L) and normal (N) birth weight pigs.

	L					*P value*		
mRNA molecules^1^	75 d	98 d	104 d	133 d	SE	75 v. 98 d	98 v. 104 d	104 v. 133 d
Lipid Uptake / Transport								
*FABP3*	-0.30	0.46	-0.09	-0.12	0.257	0.15	0.16	1.00
*FABP4*	-0.35	0.40	0.02	0.22	0.210	0.06	0.35	0.81
*LPL*	0.15	-0.09	0.07	-0.03	0.055	0.01	0.05	0.45
Lipid Synthesis								
*DGAT1*	-0.40	0.08	-0.19	0.47	0.151	0.10	0.37	*<0*.*01*
*MOGAT1*	-0.01	-0.07	0.25	-0.19	0.163	0.99	0.26	0.07
*PLIN1*	-0.11	0.09	-0.11	0.21	0.111	0.56	0.41	0.08
*PLIN2*	-0.33	0.04	-0.13	0.42	0.102	0.06	0.48	*<0*.*01*
Lipolysis								
*ATGL*	-0.15	0.22	-0.12	0.13	0.093	0.03	0.01	0.10
*HSL*	-1.19	0.51	-0.71	1.44	0.212	*<0*.*01*	<0.01	*<0*.*01*
*MGLL*	-0.32	0.13	-0.04	0.29	0.112	0.03	0.48	0.04
Nuclear Receptors								
*NR4A1*	0.59	-0.19	0.66	-0.87	0.193	0.03	<0.01	*<0*.*01*
Energy Regulation								
*PGC1*	0.03	0.18	0.13	-0.40	0.196	0.95	1.00	0.08
	N					*P value*		
mRNA molecules^1^	75 d	98 d	104 d	133 d	SE	75 v. 98 d	98 v. 104 d	104 v. 133 d
Lipid Transport								
*FABP3*	-0.11	0.37	-0.28	-0.07	0.257	0.02	0.07	0.86
*FABP4*	-0.45	0.43	-0.20	0.08	0.210	0.51	0.04	0.61
Lipid Synthesis								
*DGAT1*	-0.55	0.03	-0.02	0.69	0.151	0.03	0.99	*<0*.*01*
*PLIN2*	-0.39	0.09	-0.09	0.48	0.102	0.01	0.43	*<0*.*01*
Lipolysis								
*ATGL*	-0.19	0.10	-0.17	0.22	0.093	0.13	0.07	*<0*.*01*
*HSL*	-1.34	0.57	-0.60	1.44	0.212	*<0*.*01*	*<0*.*01*	*<0*.*01*
*MGLL*	-0.41	-0.03	-0.09	0.45	0.112	0.08	0.93	*<0*.*01*
Beta-oxidation								
*ACAA2*	-0.15	0.26	-0.27	-0.09	0.220	0.52	0.10	0.87
Nuclear Receptors								
*NR4A1*	0.53	-0.35	0.24	-0.66	0.193	0.01	0.04	*<0*.*01*
Energy Regulation								
*PGC1*	0.12	-0.11	0.27	-0.32	0.196	0.83	0.34	0.05

Data are presented as least square means and their standard error (SE). SE; the largest value is shown

^1^mRNA abundance values are Calibrated Normalized Relative Quantity (CNRQ) generated in qBASEplus 2.0. Only molecules with *P* values ≤ 0.15 are shown

Analysis of data from the birth weight groups showed that pre-dietary change the abundance of *LPL* (*P =* 0.02) and *ACACA* (lipid synthesis; *P =* 0.13) mRNA were higher and *HADH* (Hydroxyacyl-CoA dehydrogenase: beta-oxidation; *P =* 0.10) tended to be lower in the ST of L pigs (Table 6). Post-dietary change *LPL* abundance trended lower (*P =* 0.14) and *MGLL* (*P =* 0.10) trended higher in the ST of L pigs (Table 6). These changes indicate that, in response to the dietary change, L pigs lowered FA uptake and malonyl-CoA synthesis, leading to increased lipolysis.

**Table 6:** The effect of diet (75, 98 days of age (d)) and age (98, 104 and 133 d; post-dietary fat reduction) on the abundance of semitendinosus mRNA associated with lipid metabolism (lipid synthesis, lipolysis and beta-oxidation). Comparison between low (L) and normal (N) birth weight pigs.

mRNA	75 d				98 d				104 d				133 d			
molecules^1^	L	N	SE	*P value*	L	N	SE	*P value*	L	N	SE	*P value*	L	N	SE	*P value*
Lipid Uptake																
*LPL*	0.15	0.03	0.055	0.02	-0.09	-0.02	0.044	0.14	0.07	-0.06	0.043	0.02	-0.03	-0.01	0.048	0.80
Lipid Synthesis																
*ACACA*	0.17	0.01	0.116	0.13	-0.09	-0.12	0.094	0.79	0.03	-0.10	0.092	0.23	0.00	0.00	0.102	1.00
*DGAT1*	-0.40	-0.55	0.151	0.30	0.08	0.03	0.116	0.75	-0.19	-0.02	0.124	0.26	0.47	0.69	0.126	0.11
*MOGAT1*	-0.01	-0.10	0.163	0.45	-0.07	-0.04	0.131	0.81	0.25	-0.05	0.126	0.03	-0.19	0.07	0.143	0.06
Lipolysis																
*MGLL*	-0.32	-0.41	0.112	0.33	0.13	-0.03	0.090	0.10	-0.04	-0.09	0.086	0.57	0.29	0.45	0.099	0.08
Beta Oxidation																
*ACAA2*	-0.23	-0.15	0.220	0.60	0.38	0.26	0.174	0.46	0.01	-0.27	0.164	0.08	-0.02	-0.09	0.193	0.65
*CPT1B*	0.12	0.07	0.282	0.85	0.32	0.19	0.227	0.61	0.05	-0.33	0.219	0.12	-0.41	-0.14	0.247	0.27
*HADH*	-0.20	0.05	0.252	0.10	0.29	0.19	0.192	0.53	0.11	-0.22	0.181	0.04	-0.16	-0.09	0.220	0.68
Nuclear Receptors																
*NR4A1*	0.59	0.53	0.193	0.71	-0.19	-0.35	0.156	0.35	0.66	0.24	0.152	0.02	-0.87	-0.66	0.170	0.25
*PPARA*	0.02	-0.06	0.099	0.30	0.03	0.03	0.080	0.96	-0.03	-0.03	0.077	0.99	-0.03	0.15	0.087	0.04

Data are presented as least square means and their standard error (SE).

^1^mRNA abundance values are Calibrated Normalized Relative Quantity (CNRQ) generated in qBASEplus 2.0. Only molecules with *P* values ≤ 0.15 are shown

Pre-dietary fat reduction, it appears that the ST of L pigs have greater FA uptake, as evidenced by the higher abundance of *LPL* mRNA. However, at 98 d, the abundance of *LPL* mRNA was lower in the ST of L pigs. These changes were also observed in the LD, suggesting that both muscles in L pigs were more sensitive to the dietary change at 80 d, potentially forcing them to reduce their reliance on FA-resources as a source of energy. In the ST of L pigs, this theory is supported by the higher *ACACA* and lower *HADH* mRNA abundance at pre-reduction in dietary fat and higher *MGLL* mRNA post-fat reduction. Acetyl-CoA carboxylase 1 is an enzyme that converts acetyl-CoA released by the mitochondria to malonyl-CoA, the rate-limiting step in FA synthesis from the mitochondria and is transcriptionally regulated [45]. The concentration of malonyl-CoA determines the switch between FA synthesis and oxidation, lower concentrations shift towards beta-oxidation, whilst higher lead to increased fatty acid synthesis [45]. A higher abundance of *ACACA* mRNA has been shown to correlate with increased concentrations of malonyl-CoA, which inhibits the first step of beta-oxidation, and has been associated with lower *HADH* mRNA abundance. Monoglyceride Lipase (MGLL) is a transcriptionally regulated gene whose protein product is the rate-limiting enzyme for lipolysis, where it is responsible for the conversion of MAG to free FA and glycerol [46]. We propose that the higher abundance of *MGLL* mRNA in the ST of L pigs was in response to the reduced ability to uptake FA when dietary fat was reduced.

### Semitendinosus, post diet adaptation

At 104 d, *ATGL* and *HSL* mRNA were lower (lipolysis; L: *P =* 0.01, *P <* 0.01; N: *P =* 0.07, *P* < 0.01) and *NR4A1* (nuclear receptor; L: *P <* 0.01; N: *P =* 0.04) was higher in both L and N pigs, compared to 98 d (Table 5). In L pigs *LPL* (lipid uptake; *P =* 0.05) was higher, and in N pigs *FABP3*, *FABP4* (*P =* 0.07, *P =* 0.04) and *ACAA2* (Acetyl-CoA acyltransferase 2: beta-oxidation; *P =* 0.10) mRNA were lower (Table 5). Analysis of the observed changes suggest that whilst lipolysis appears to have reduced in both L and N pigs over time, L pigs have potentially increased FA uptake whilst N pigs may have reduced beta-oxidation. At 133d, *ATGL*, *HSL*, *MGLL* (lipolysis; L: *P =* 0.10, *P <* 0.01, *P =* 0.04; N: *P <* 0.01), *DGAT1*, *PLIN2* (lipid synthesis; L: *P <* 0.01; N: *P <* 0.01) mRNA were higher and *NR4A1* (nuclear receptor; L: *P <* 0.01; N: *P <* 0.01) and *PGC1* (energy regulation; L: *P =* 0.08; N: *P =* 0.05) were lower in both L and N pigs. Additionally, in L pigs *MOGAT1* (Monoacylglycerol O-acyltransferase 1) and *PLIN1* (lipid synthesis; *P =* 0.07, *P =* 0.08) trended higher at 133 d. Thus, whilst lipolysis and lipid synthesis appeared to be higher in both L and N pigs at 133 d, only L pigs appeared to increase INTMF lipid synthesis.

Birth weight group comparisons showed that at 98 d *LPL* (*P =* 0.14) mRNA trended lower and *MGLL* (*P =* 0.10) trended higher in L pigs. By 104 d, *LPL* (*P =* 0.02), *MOGAT1* (*P =* 0.03) *ACAA2*, *CPT1B*, *HADH* (beta-oxidation; *P =* 0.04, *P =* 0.08, *P =* 0.12) and *NR4A1* (nuclear receptor; *P =* 0.02) mRNA abundance was higher in L pigs (Table 6). At 131 d, the abundance of *DGAT1*, *MOGAT1* (*P =* 0.06, *P =* 0.11), *MGLL* (*P =* 0.08) tended and *PPARA* (nuclear receptor; *P =* 0.04) mRNA was lower in L pigs (Table 6). Analysis of the data indicated that L pigs transitioned from higher beta-oxidation to higher lipolysis and lipid synthesis compared to N pigs.

At 98 d, a higher abundance of MGLL, an indicator of increased lipolysis, was observed in the ST of L pigs, and by 104 d, the abundance of beta-oxidation-associated *CPT1B*, *ACAA2* and *HADH* mRNA was higher in the ST of L pigs. As previously mentioned *CPT1B* encodes the muscle specific isoform of CPT1 and is the rate-limiting step in the uptake of long chain fatty acids by the mitochondria, whilst *HADH* and *ACAA2* mRNA are part of the mitochondrial trifunctional protein, which catalyses the last steps of beta-oxidation [40]. Interestingly, these three genes are regulated by PPARA, which in our study was not different between L and N pigs. It is possible PPARA stimulation occurred at an earlier time point, or NR4A1, whose mRNA abundance was higher in L pigs, is the transcriptional regulator of *Cpt1b*, *Hadh* and *Acaa2*, as it has been shown that *Cpt1* transcription can be controlled by NR4A1 [47]. Additionally, *MOGAT1* mRNA abundance was higher in the ST of L pigs. Monoacylglycerol acyltransferase enzymes convert MAG to DAG [48], where DAG not only acts as precursor for TAG synthesis, but several studies show that elevated DAGs are associated with impaired insulin signalling and increased beta-oxidation [49], as observed in this study. By 131 d, there is no longer a difference in the abundance of beta-oxidation associated mRNA, between the ST of L and N pigs, and lipid synthesis and lipolysis appears to be repressed in L pigs, as evidenced by the reduced abundance of *PPARA* mRNA, and three of its downstream targets, *MGLL* [50], *MOGAT1* [51] and *DGAT1* [52].

### Perspectives

The lipid metabolism transcriptional profile differences observed between the skeletal muscles of L and N pigs may be explained by the influence birth weight has on muscle fibre number [6], type [53, 54] and subsequent postnatal growth [8]. L individuals have been shown to have a reduced number of secondary fibres [6] potentially resulting in fibre growth being attained earlier and energy being partitioned towards fat deposition (8). Additionally, the beta-oxidation capacity of skeletal muscle is determined by the fibre type composition, where low beta-oxidation occurs in fast-glycolytic, medium to high in fast-oxidative-glycolytic and high occurs in slow-oxidative fibres [55]. Changes in muscle fibre composition have been observed in humans [53], mice [17] and pigs [54, 56], that have been associated with increased adiposity and the development of insulin resistance.

Whilst a direct comparison of the individual mRNA molecules between LD and ST could not be performed, the associated shifts in the pathways they belong to suggest each muscle adapted differently during the change in diet and the post-diet adaptation period. Associated with the change in diet it appears both muscles reduced their reliance on FA-resources as a source of energy. However, where the LD appeared to reduce markers of lipolysis and lipid synthesis post-diet change, the ST increased them; the biology behind this remains unclear and requires further investigation. During the post-diet adaption phase, the LD appears to increase the abundance of markers of lipid synthesis (IMF and INTMF), and beta-oxidation whereas the ST has associated increases in the markers of lipid synthesis (IMF) and lipolysis. Thus, the changes in mRNA abundance from the ST match with the observed phenotype from L and N pigs [12].

## Conclusion

The results presented in this study suggest that our previous observations of low birth weight being associated with changes in whole body metabolic physiology may be linked to adaptation of skeletal muscle lipid metabolism, which shifts in response to a reduction of dietary fat in adolescent female pigs. We propose that extra- and/or intra-mitochondrial regulatory dysfunction links impaired in utero growth with future metabolic disease.

## Supporting information

S1 Table Experimental litter. (DOCX)

S2 Table Diet components, dry matter (DM), calculated crude nutrient composition, and metabolizable energy (ME), classified according to age of pigs. (DOCX)

S3 Table Experimental design. (DOCX)

S4 Table Name, symbol, accession number, primer sequence and amplicon size of genes analyzed by qPCR. (DOCX)
