# Supplementary material for: Transcript profile of skeletal muscle lipid metabolism genes affected by diet in a piglet model of low birth weight
Source: PLoS One. 2019 Oct 29;14(10):e0224484. doi: 10.1371/journal.pone.0224484 (PMC6818798; doi:10.1371/journal.pone.0224484)
Supplement: S1 Table — (DOCX) [file pone.0224484.s001.docx]

**S1 Table.** **Experimental litter characteristics**

| Age Group | No. Sows | No. Boars | Average litter size | | Litter gender (%) | | | |  | Average piglet weight (kg) | | | | | |
| --- | --- | --- | --- | --- | --- | --- | --- | --- | --- | --- | --- | --- | --- | --- | --- |
|  |  |  | Birth | post 24-h | Birth | | post 24-h | |  | Birth | | Weaning | | Euthanasia | |
|  |  |  |  |  | Male | Female | Male | Female |  | N | L | N | L | N | L |
| 76 | 8 | 4 | 15 ± 3 | 13 ± 2 | 46 | 54 | 42 | 58 |  | 1.53 ± 0.12 | 1.12 ± 0.09 | 8.31 ± 1.86 | 6.15 ± 1.02 | 24.19 ± 3.27 | 20.05 ± 2.63 |
| 99 | 9 | 6 | 16 ± 2 | 13 ± 2 | 43 | 57 | 40 | 60 |  | 1.57 ± 0.13 | 1.09 ± 0.09 | 8.24 ± 1.18 | 6.19 ± 1.03 | 35.20 ± 5.52 | 33.03 ± 2.63 |
| 104 | 9 | 4 | 16 ± 3 | 13 ± 1 | 42 | 58 | 43 | 57 |  | 1.45 ± 0.06 | 1.08 ± 0.09 | 8.02 ± 1.74 | 6.54 ± 0.92 | 42.70 ± 5.30 | 38.16 ± 6.29 |
| 133 | 8 | 4 | 15 ± 2 | 14 ± 2 | 41 | 59 | 41 | 59 |  | 1.53 ± 0.10 | 1.08 ± 0.09 | 9.08 ± 1.52 | 6.88 ± 0.89 | 69.46 ± 9.39 | 67.48 ± 4.88 |
