# Supplementary material for: Transcript profile of skeletal muscle lipid metabolism genes affected by diet in a piglet model of low birth weight
Source: PLoS One. 2019 Oct 29;14(10):e0224484. doi: 10.1371/journal.pone.0224484 (PMC6818798; doi:10.1371/journal.pone.0224484)
Supplement: S2 Table — (DOCX) [file pone.0224484.s002.docx]

**S2 Table.** **Diet components, dry matter (DM), calculated crude nutrient composition, and metabolizable energy (ME), classified according to age of pigs**

|  | Age (day) | | |
| --- | --- | --- | --- |
|  | 28-41 | 42-79 | 80-131 |
| Diet components (weight % of daily feed allowance) | | | |
| Starter feed^1^ | 50 | 46.5 | - |
| Grower feed^2^ | - | - | 68.5 |
| Oat flakes^3^ | 30 | 33.8 | 20.7 |
| Sucrose^4^ | 20 | 19.7 | 10.8 |
| DM (%) | 98 | 98 | 98 |
| Crude nutrients (g/kg) | | | |
| Crude ash | 35 | 34 | 44 |
| Crude protein | 162 | 159 | 165 |
| Crude fat | 94 | 92 | 38 |
| Carbohydrates | 604 | 612 | 641 |
| Crude fiber | 16 | 16 | 39 |
| Starch | 314 | 330 | 440 |
| Sugar | 278 | 270 | 148 |
| ME^5^ (MJ/kg DM) | 16.9 | 16.9 | 15.2 |

^1^ ‘Baby Crisb,’ Bergophor, Hohburg Mineralfutter GmbH, Hohburg, Germany

^2^ ‘Vormast CaFo TOP,’ Trede und von Pein GmbH, Itzehoe, Germany; during food restriction diet was devoid of sugar and oat flakes

^3^ Holstenmuhle W. Smidt & Co. KG, Lu¨beck, Germany

^4^ Nordzucker, Braunschweig, Germany

^5^ Calculated according to the GfE (2008): ME (MJ/kg DM) = 0.021503 × crude protein (g/kg DM) + 0.032497 × crude fat (g/kg DM) – 0.021071 × crude fiber (g/kg DM) + 0.016309 × starch (g/kg DM) + 0.014701 × organic residue (g/kg DM)
