# Supplementary figures and images for: Transcript profile of skeletal muscle lipid metabolism genes affected by diet in a piglet model of low birth weight
Source: PLoS One. 2019 Oct 29;14(10):e0224484. doi: 10.1371/journal.pone.0224484 (PMC6818798; doi:10.1371/journal.pone.0224484)

**S3 Table. Experimental design**

**
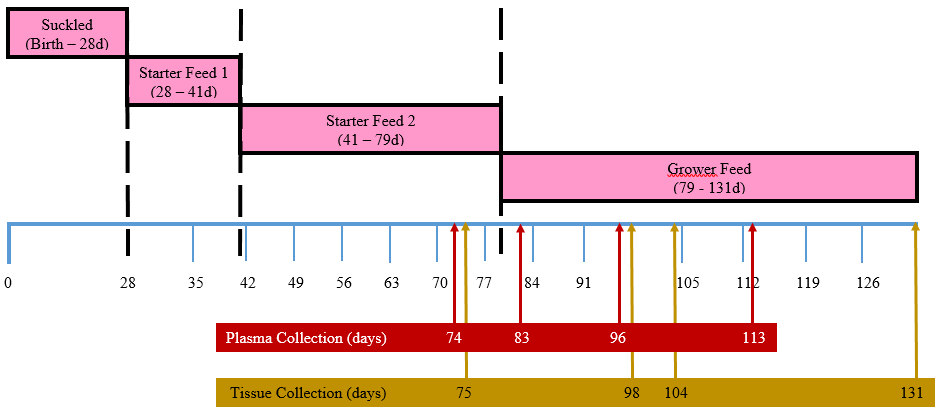
**

Supplement: S3 Table — (DOCX) [file pone.0224484.s003.docx]
