# Supplementary material for: Transcript profile of skeletal muscle lipid metabolism genes affected by diet in a piglet model of low birth weight
Source: PLoS One. 2019 Oct 29;14(10):e0224484. doi: 10.1371/journal.pone.0224484 (PMC6818798; doi:10.1371/journal.pone.0224484)
Supplement: S4 Table — (DOCX) [file pone.0224484.s004.docx]

**S4 Table.** **Name, symbol, accession number, primer sequence and amplicon size of genes analyzed by qPCR.**

| Gene name | Symbol | Accession no. | Hybridization: Sequence (5´ - 3´) | Amplicon size (bp) | PCR Efficiency^1^ |
| --- | --- | --- | --- | --- | --- |
| Reference |  |  |  |  |  |
| Hypoxanthine Phosphoribosyltransferase 1 | HPRT1 | NM_001032376.2 | GGACTTGAATCATGTTTGTG | 91 | 1.90 |
|  |  |  | CAGATGTTTCCAAACTCAAC |  |  |
| Heat Shock Protein 90kDa Alpha, Class B | HSPCB | XM_005666063.1 | AGATCACCTGGCAGTCAAGC | 198 | 1.89 |
|  |  |  | CCGCGGATGAAGTTGAGGTA |  |  |
| Proteasome 26S Subunit, ATPase 3 | PSMC3 | XM_005660922.1 | GCAAGTGTGCAGTGATCAAAA | 112 | 1.91 |
|  |  |  | TCTTTGTTCACACCCACCAA |  |  |
| Ribosomal Protein S18 | RPS18 | NM213940.1 | GGATGTGAAGGATGGGAAGTACA | 72 | 1.94 |
|  |  |  | TCCAAGTCTTCACGGAGTTTGTT |  |  |
| Topoisomerase (DNA) II Beta | TOP2B | NM_001258386.1 | CCCAGTTGGCTGGATCTGTT | 182 | 1.84 |
|  |  |  | ATAACGAGGGCTTGCAGCAT |  |  |
| Lipid Uptake / Transport |  |  |  |  |  |
| Lipoprotein lipase | LPL | NM_214286.1 | CAATGGAGGCACTTTCCAAC | 70 | 1.89 |
|  |  |  | AAGGCCTCTCTCTGCAATCA |  |  |
| Fatty acid-binding protein 3 | FABP3 | NM_001099931.1 | AAGCACCTTCAAGAGCACAGA | 92 | 1.90 |
|  |  |  | TCACAATGGACTTGACCTTCC |  |  |
| Fatty acid binding protein 4 | FABP4 | NM_001002817.1 | GAAGTGGGAGTGGGCTTTG | 63 | 1.91 |
|  |  |  | GATGATCAGGTTGGGTTTGG |  |  |
| Lipid Synthesis |  |  |  |  |  |
| Diacylglycerols acyltransferase 1 | DGAT1 | NM_214051.1 | GGAGATGCTGTTCCTCATCC | 87 | 1.90 |
|  |  |  | TTGAAGGGCTTCATGGAGTT |  |  |
| Diacylgycerol acyltransferase 2 | DGAT2 | NM_001160080.1 | CCAGCAGGTGATCTTTGAGG | 76 | 1.91 |
|  |  |  | GGCGAAGCCAATGTACTTCT |  |  |
| Fatty acid synthase | FASN | NM_001099930.1 | CATGGAGCAAGGCAAGGT | 89 | 1.91 |
|  |  |  | TCTGCGTGATCAGGTCCAC |  |  |
| Glycerol-3-phosphate acyltransferase 1 | GPAT1 | XM_001927875.2 | TGCTGATCAACTGCTTGGAC | 190 | 1.76 |
|  |  |  | CACTCACCCCATTCCTCACT |  |  |
| Glycerol-3-phosphate acyltransferase 2 | GPAT2 | XM_005671462.1 | “Forward primer from GPAT1” | 243 | 1.77 |
|  |  |  | AAGTACGGGCAGCTGCAGAG |  |  |
| Glycerol-3-phosphate acyltransferase 3 | GPAT3 | XM_005671463.1 | “Forward primer from GPAT1” | 167 | 1.79 |
|  |  |  | CTATCCAATCCTCCCAGCAA |  |  |
| Monoacylglycerol O-acyltransferase 1 | MOGAT1 | XM_003133676.2 | CAAGGAACTGTTTCCTGGCT | 148 | 1.93 |
|  |  |  | ACCTCCTTCCTTTCGTAGCA |  |  |
| Monoacylglycerol O-acyltransferase 2 | MOGAT2 | NM_001167651.1 | CCCTTCTTCAGGGATTACATCA | 63 | 1.87 |
|  |  |  | AGCAGCGCTCTCCTTGTCT |  |  |
| Perilipin 1 | PLIN1 | NM_001038638.1 | CTGACTTTGCTGGATGGAGA | 112 | 1.91 |
|  |  |  | TGCTGGTGTAGGTCTTCTGG |  |  |
| Perilipin 2 | PLIN2 | NM_214200.2 | GTGTGAGATGGCAGAGAAGG | 117 | 1.86 |
|  |  |  | TTACAGGCGTAAGTGTTGGC |  |  |
| Stearoyl-CoA desaturase | SCD | NM_213781.1 | CTGGCTTATGACCGGAAGAA | 96 | 1.90 |
|  |  |  | ACCCCAAACTCAGCCACTC |  |  |
| Lipolysis |  |  |  |  |  |
| Adipose triglyceride lipase | ATGL | EF583921.1 | CAGCTGCCCACGAGTGATA | 109 | 1-90 |
|  |  |  | GGTTGGACAGGGTGCTCA |  |  |
| Hormone-sensitive lipase | HSL | NM_214315.1 | CCACGAGCCTTACCTCAAGA | 73 | 1.88 |
|  |  |  | CGCTAGGGAGTAGTCGATGG |  |  |
| Monoglyceride Lipase | MGLL | NM_001143718.1 | CACAGCTCCCAGTCTTCCTT | 132 | 1.94 |
|  |  |  | CAGACTCAGGACTGGCAAGA |  |  |
| Beta Oxidation |  |  |  |  |  |
| Acetyl-CoA acyltransferase 2 | ACAA2 | NM_001167638.1 | GGCACTGAAGAAAGCAGGAC | 238 | 1.90 |
|  |  |  | GGCTGATCCCACAGCATATT |  |  |
| Acetyl-CoA carboxylase Alpha | ACACA | NM_001114269.1 | GGTTATGTGAAGGATGTGGATG | 75 | 1.89 |
|  |  |  | TGAGGCCTTGATCATTACTGG |  |  |
| Acyl-CoA dehydrogenase. long chain | ACADL | NM_213897.1 | CATGAAACGAAACGTCTGGA | 159 | 1.88 |
|  |  |  | AACTCGGGCATCCACATAAG |  |  |
| Carnitine palmitoyltransferase 1A | CPT1A | NM_001129805.1 | AAGGTGCTGCTCTCCTACCA | 204 | 1.86 |
|  |  |  | CATCAGAGGCTTCACGGATT |  |  |
| Carnitine palmitoyltransferase 1B | CPT1B | NM_001007191.1 | AACAGCGGGTTCCTCCTACT | 196 | 1.90 |
|  |  |  | GGAAAGCAGCAGTTTCAAGG |  |  |
| Hydroxyacyl-CoA dehydrogenase | HADH | NM_214331.1 | GAGGAAAGCCTTCGGAAAGT | 175 | 1.91 |
|  |  |  | CGCTCTTCACCTTCAGGTTC |  |  |
| Nuclear Receptors |  |  |  |  |  |
| Nuclear Receptor Subfamily 4, Group A Member 1 | NR4A1 | FJ548761.1 | CCAGCACTTCCAAACTGGAC | 109 | 1.91 |
|  |  |  | GAACCCGAGAGCAGGTCAT |  |  |
| Peroxisome proliferator-activated receptor alpha | PPARA | NM_001044526.1 | TGCCAGTATTGTCGTTTCCA | 219 | 1.92 |
|  |  |  | GGCCTTGACCTTGTTCATGT |  |  |
| Peroxisome proliferator-activated receptor gamma | PPARG | AF059245.1 | CATGCTGTCATGGGTGAAAC | 188 | 1.89 |
|  |  |  | TCAAAGGAGTGGGAGTGGTC |  |  |
| Energy Regulation  Peroxisome proliferator-activated receptor gamma coactivator 1 | PGC-1 | NM_213963.1 | CCGAGAATTCATGGAGCAAT | 162 | 1.91 |
|  |  |  | TGGAGGTGCACTTGTCTCTG |  |  |

^1^ Calculated using LinRegPCR
